# Redescription of *Pseudopoda taibaischana* (Araneae, Sparassidae), with the first description of the female

**DOI:** 10.3897/zookeys.991.56969

**Published:** 2020-11-11

**Authors:** Li-Jun Gong, Yang Zhong

**Affiliations:** 1 Hubei Key Laboratory of Radiation Chemistry and Functional Materials, School of Nuclear Technology and Chemistry & Biology, Hubei University of Science and Technology, Xianning 437100, Hubei, China Hubei University of Science and Technology Hubei China

**Keywords:** Biodiversity, huntsman spiders, Shaanxi, taxonomy

## Abstract

*Pseudopoda taibaischana* Jäger, 2001 (Sparassidae) is redescribed based on new material from the type locality in Taibaishan Nation Forest Park of Shaanxi Province, China. The female is described and illustrated for the first time, and a redescription is provided for the male.

## Introduction

The genus *Pseudopoda* was established by Jäger (2000) to include nine species previously assigned to *Heteropoda*: *P. casaria* (Simon, 1897), *P. exigua* (Fox, 1938), *P. exiguoides* (Song & Zhu, 1999), *P. grahami* (Fox, 1936), *P. lushanensis* (Wang, 1990), *P. prompta* (O. Pickard-Cambridge, 1885), *P. virgata* (Fox, 1936), *P. zhangmuensis* (Hu & Li, 1987), and *P. zhejiangensis* (Zhang & Kim, 1996). Currently, *Pseudopoda* is the third largest genus of the subfamily Heteropodinae, and includes 142 species. Members of this genus are known from Bhutan, China, India, Indonesia, Japan, Laos, Myanmar, Nepal, Pakistan, Thailand, and Vietnam (World Spider Catalog 2020). From China, 63 species are known; among them, seven species are only known from females and eleven only from males (World Spider Catalog 2020). *Pseudopoda taibaischana* Jäger, 2001 was first described based on one male specimen from Taibaishan National Forest Park of Shaanxi Province, China (Jäger 2001). Recently, new material of both sexes was collected from the type locality of this species, enabling us to describe the female for the first time in this paper.

## Materials and methods

Specimens were examined and measured with a Leica M205C stereomicroscope. The points arising from the tegular appendages are listed as clock-positions from the left bulb in ventral view. Male palps were examined after dissection and detachment. The epigynes were examined and illustrated after dissection: they were removed and cleared in warm lactic acid before illustration. The vulva was photographed after being embedded in Arabic gum. All photographs were taken with a Leica DFC450 digital camera attached to a Leica M205C stereomicroscope, with 10–20 photographs taken in different focal planes and combined using the image stacking software Leica LAS. Images were edited using Adobe Photoshop CC 2015.

Leg measurements are listed as: total length (femur, patella, tibia, metatarsus, tarsus). The number of spines is listed for each segment in the following order: prolateral, dorsal, retrolateral, ventral (in femora and patellae, ventral spines are absent, and the fourth digit is omitted in the spination formula).

Abbreviations used in the text and figures are given below:

**SMF**Senckenberg Research Institute and Museum, Frankfurt, Germany (P. Jäger);

**HUST**School of Nuclear Technology and Chemistry & Biology, Hubei University of Science and Technology, Xianning, Hubei, China (Y. Zhong);

**ALE** anterior lateral eye;

**AME** anterior median eye;

**AW** anterior width of carapace;

**C** conductor;

**CO** copulatory opening;

**CH** clypeus height;

**E** embolus;

**EP** embolic projection;

**FD** fertilization duct;

**FE** femur;

**FW** first winding;

**LL** lateral lobes;

**Mt** metatarsus;

**OL** opisthosoma length;

**OW** opisthosoma width;

**Pa** patella;

**PI** posterior incision of LL;

**PL** carapace length;

**PLE** posterior lateral eyes;

**PME** posterior median eyes;

**Pp** palp;

**PP** posterior part of spermathecae;

**PW** carapace width;

**RTA** retrolateral tibial apophysis;

**S** spermathecae;

**T** tegulum;

**Ta** tarsus;

**Ti** tibia. I, II, III, IV–legs I to IV.

## Taxonomy

**Family Sparassidae Bertkau, 1872**

### Subfamily Heteropodinae Thorell, 1873

#### Genus Pseudopoda

Taxon classificationAnimaliaAraneaeSparassidae

Jäger, 2000

AF89897A-1FFE-5E55-8089-359AB924931C

##### Type species.

*Sarotes promptus* O. Pickard-Cambridge, 1885.

##### Diagnosis.

See Jäger (2000) and Jiang et al. (2018).

##### Composition.

*P. daliensis*-group (*P. anguilliformis*Zhang et al., 2017, *P. peronata*Zhang et al., 2017, *P. sicyoidea*Zhang et al., 2017, *P. daliensis* Jäger & Vedel, 2007, *P. kunmingensis* Sun & Zhang, 2012), *P. diversipunctata*-group (*P. diversipunctata* Jäger, 2001, *P. intermedia* Jäger, 2001, *P. marsupia* (Wang, 1991)), *P. latembola*-group (*P. albolineata* Jäger, 2001, *P. alta* Jäger, 2001, *P. chauki* Jäger, 2001, *P. everesta* Jäger, 2001, *P. latembola* Jäger, 2001, *P. monticola* Jäger, 2001, *P. sinopodoides* Jäger, 2001), *P. martensi*-group (*P. chulingensis* Jäger, 2001, *P. dhulensis* Jäger, 2001, *P. gogona* Jäger, 2001, *P. hyatti* Jäger, 2001, *P. kalinchoka* Jäger, 2001, *P. khimtensis* Jäger, 2001, *P. martensi* Jäger, 2001, *P. martinae* Jäger, 2001, *P. megalopora* Jäger, 2001, *P. platembola* Jäger, 2001, *P. tinjura* Jäger, 2001, *P. varia* Jäger, 2001, *P. virgata* (Fox, 1936)), *P. parvipunctata*-group (*P. biapicata* Jäger, 2001, *P. dao* Jäger, 2001, *P. jirensis* Jäger, 2001, *P. parvipunctata* Jäger, 2001, *P. schawalleri* Jäger, 2001, *P. thorelli* Jäger, 2001, *P. triapicata* Jäger, 2001, *P. lushanensis* (Wang, 1990)), *P. prompta*-group (*P. brauni* Jäger, 2001, *P. cuneata* Jäger, 2001, *P. grasshoffi* Jäger, 2001, *P. huberti* Jäger, 2001, *P. marmorea* Jäger, 2001, *P. trisuliensis* Jäger, 2001, *P. casaria* (Simon, 1897), *P. prompta* (O. Pickard-Cambridge, 1885), *P. zhangmuensis* (Hu & Li, 1987)), *P. schwendingeri*-group (*P. hirsuta* Jäger, 2001, *P. schwendingeri* Jäger, 2001), and *P. signata*-group (*P. bibulba* (Xu & Yin, 2000), *P. physematosa*Zhang et al., 2019, *P. semilunata*Zhang et al., 2019, *P. signata* Jäger, 2001, *P. wu* Jäger, Li & Krehenwinkel, 2015, *P. yinae* Jäger & Vedel, 2007, *P. yunnanensis* (Yang & Hu, 2001)) and 88 other species that have not yet been grouped.

#### Pseudopoda taibaischana

Taxon classificationAnimaliaAraneaeSparassidae

Jäger, 2001

5C2CF12F-ADC0-5E7C-86AF-60EF1530030D

**Figure 1.**
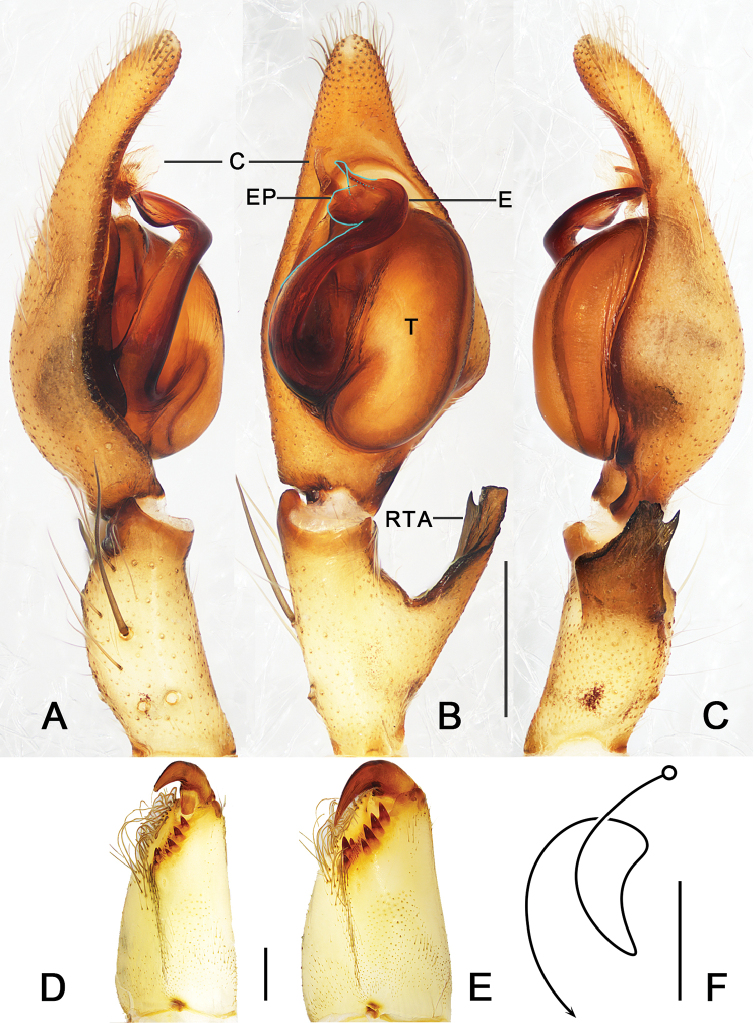
*Pseudopoda taibaischana* Jäger, 2001 **A–C** left male palp (**A** prolateral view **B** ventral view **C** retrolateral view) **D, E** cheliceral dentition, ventral view (**D** male **E** female) **F** schematic course of internal duct system. Abbreviations: C–conductor, E–embolus, EP–embolic projection, RTA–retrolateral tibial apophysis, T–tegulum. Scale bars: 0.5 mm.

**Figure 2.**
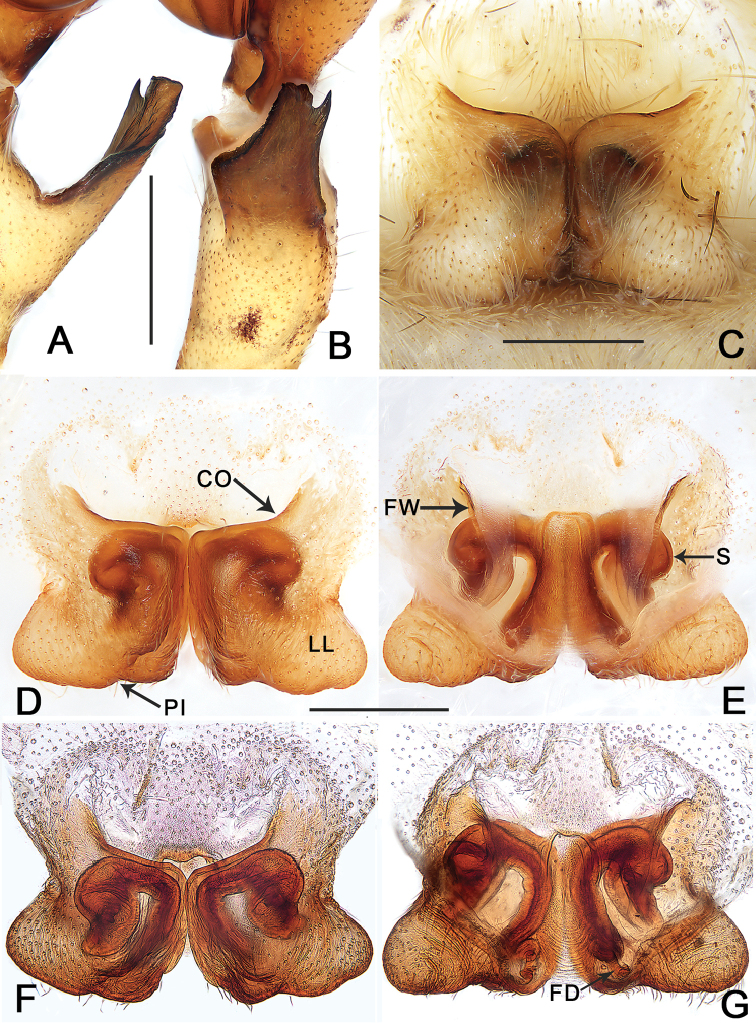
*Pseudopoda taibaischana* Jäger, 2001 **A, B** Left male palpal tibia (**A** ventral view **B** retrolateral view) **C** epigyne, intact **D** epigyne, cleared **E** vulva, cleared **F** epigyne, cleared and embedded in Arabic gum **G** vulva, cleared and embedded in Arabic gum (**C, D, F** ventral view **E, G** dorsal view). Abbreviations: CO–copulatory opening, FD–fertilization duct, FW–first winding, LL–lateral lobes, PI–posterior incision of LL, S–spermathecae. Scale bars: 0.5 mm.

**Figure 3.**
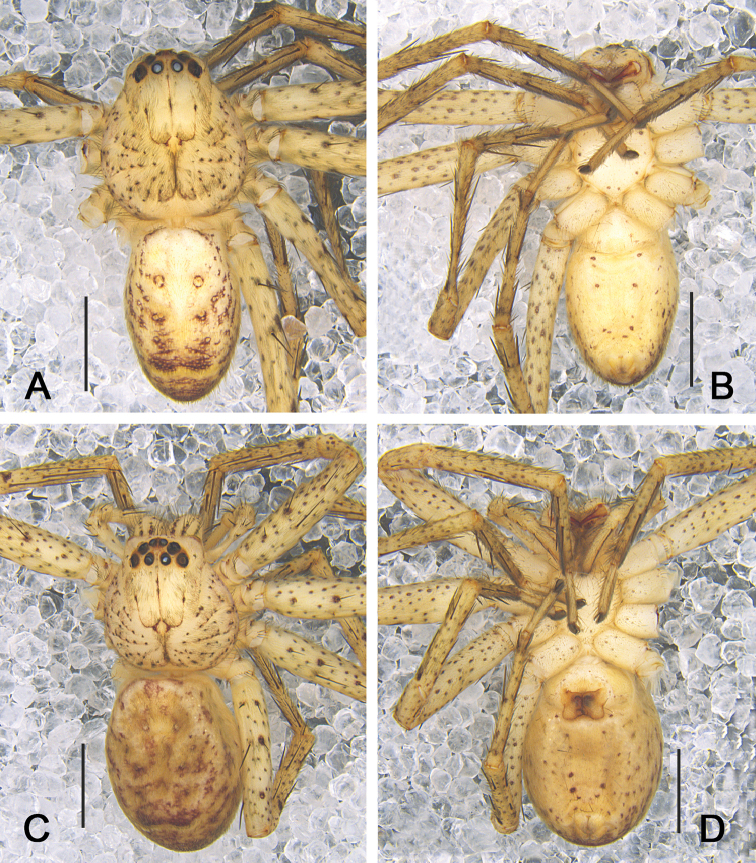
*Pseudopoda taibaischana* Jäger, 2001 **A, B** male habitus (**A** dorsal view **B** ventral view) **C, D** female habitus (**C** dorsal view **D** ventral view). Scale bars: 2 mm.

**Figure 4.**
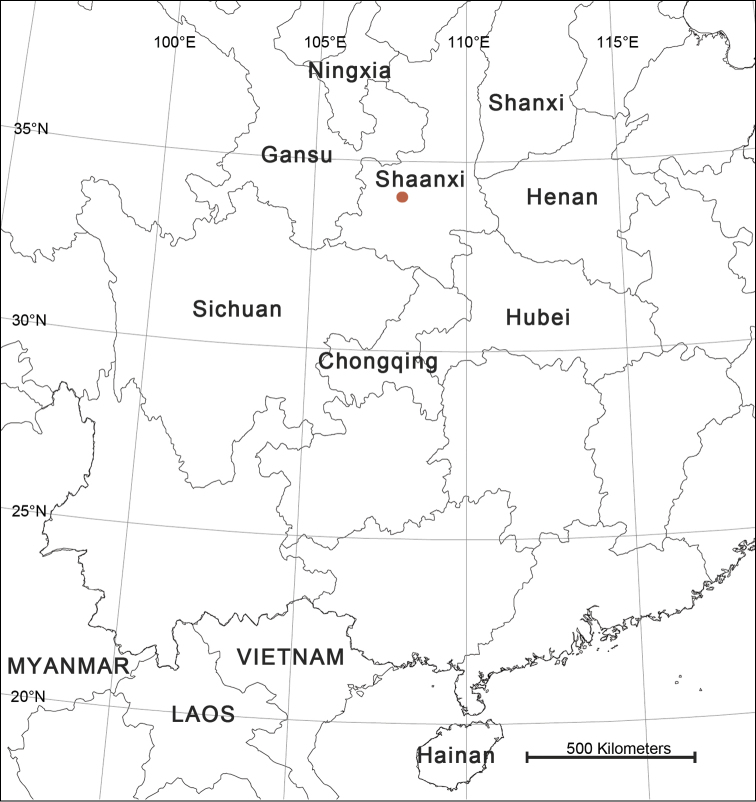
Collection localities of *Pseudopoda taibaischana* in Shaanxi Province, China.

Figures 1 , 2 , 3 , 4

Pseudopoda taibaischana Jäger, 2001: 86, figs 47a–e (holotype male from Taibaishan National Forest Park of Shaanxi Province, deposited in SMF PJ1056)

##### Material examined.

2♂, 10♀ (HUST 0001), Shaanxi Province, Baoji City, Taibaishan National Forest Park; 34.05°N, 107.87°E; alt. 1438 m; 20.VII. 2019, Y. Zhong leg.

##### Diagnosis.

This species resembles *Pseudopoda cangshana* Jäger & Vedel, 2007 (Jäger and Vedel 2007: figs 66–68, 70–72) in having the embolus strongly S-shaped, proximal part of embolus visible, and lateral loops of internal duct system extending laterally beyond its first winding, but can be distinguished from the latter by the following characters: 1, male palp with laminar and rounded embolic projection (absent in *P. cangshana*); 2, tip of RTA with distinct triangular extension dorsally (absent in *P. cangshana*); 3, female epigyne with converging part of anterior margins of lateral lobes T-shaped (Y-shaped in *P. cangshana*); 4, female vulva with loops of internal duct system distinctly curved in ventral view (not curved in *P. cangshana*) (Figs 1, 2).

##### Description.

**Male.**PL 3.7, PW 2.8, AW 2.0, OL 4.0, OW 3.2. Eyes and interdistances: AME 0.20, ALE 0.25, PME 0.21, PLE 0.27, AME–AME 0.18, AME–ALE 0.07, PME–PME 0.24, PME–PLE 0.30, AME–PME 0.32, ALE–PLE 0.27, CHAME 0.30, CHALE 0.28. Spination: Palp: 131, 101, 2101; Fe: I–III 323, IV 331; Pa: I–IV 001; Ti: I–II 2026, III–IV 2126; Mt: I–II 2024, III 3024, IV 3036. Measurements of palp and legs: Palp 6.3 (2.0, 1.1, 1.2, –, 2.0), I 17.9 (4.7, 2.0, 5.4, 4.2, 1.6), II 19.4 (5.4, 2.1, 5.8, 4.3, 1.8), III 14.9 (4.4, 1.6, 4.2, 3.3, 1.4), IV 18.2 (5.2, 1.7, 4.7, 4.9, 1.7). Leg formula: 2-4-1-3. Cheliceral furrow with three anterior and four posterior teeth, each tooth with 22 denticles (Fig. 1D). Carapace yellowish brown, with fovea slightly darker and bearing more spots. Chelicerae deep reddish brown. Sternum pale yellow, with small and irregular spots. Legs yellowish brown, with medium-sized spots and slightly larger spine patches. Abdomen yellowish brown dorsally, with three pairs of dark patches laterally and an irregular pattern in posterior half; ventrally yellowish brown with small and irregular patches (Fig. 3A, B).

Palp as in diagnosis. Cymbium longer than tibia. Embolus arising from tegulum at 8 o’clock position, embolic projection making the tip of embolus look somewhat incised. Conductor curved, arising from an 11 o’clock position. Spermophor visible and slightly curved in retrolateral view. RTA arising medially from tibia, with only one apex, broad in retrolateral view (Figs 1, 2A, B).

**Female.**PL 3.6, PW 3.2, AW 2.3, OL 4.7, OW 3.4. Eyes and interdistances: AME 0.17, ALE 0.23, PME 0.20, PLE 0.26, AME–AME 0.16, AME–ALE 0.10, PME–PME 0.23, PME–PLE 0.30, AME–PME 0.33, ALE–PLE 0.28, CHAME 0.35, CHALE 0.31. Spination: Palp: 131, 101, 1014, 2121; Fe: I–III 323, IV 331; Pa: I–IV 001; Ti: I 2026, III–IV 2126; Mt: I–II 2024, III 3025, IV 3036. Measurements of palp and legs: Palp 4.9 (1.6, 0.7, 1.0, –, 1.6), I 12.8 (3.8, 1.7, 3.3, 2.8, 1.2), II 14.0 (4.3, 1.3, 3.7, 3.4, 1.3), III 11.6 (3.5, 1.4, 2.8, 2.9, 1.0), IV 13.3 (4.0, 1.3, 3.3, 3.5, 1.2). Leg formula: 2-4-1-3. Cheliceral furrow with three anterior and four posterior teeth, each tooth with 32 denticles (Fig. 1E).

Epigynal field only slightly wider than long, with very short anterior bands or without such bands. Anterior margins of lateral lobes bent anteriorly at their lateral ends. Posterior incision of lateral lobe distinct, near the posterior meeting point of lateral lobes. Base of internal duct system distinctly extending laterally beyond first winding (Fig. 2C–G).

Coloration in ethanol: as in male, but generally darker, abdomen with more spots ventrally (Fig. 3C, D).

##### Distribution.

China (Shaanxi Province) (Fig. 4).

## Supplementary Material

XML Treatment for Pseudopoda

XML Treatment for Pseudopoda taibaischana
